# Case Report: A rare but critical complication in patients with lumbar infection combined with cauda equina syndrome—sacrococcygeal pressure sores

**DOI:** 10.3389/fmed.2026.1776438

**Published:** 2026-03-06

**Authors:** Zhen Jia, Bo Yu, Di Jiang, Zhengqi Chang, Shiyong Wan

**Affiliations:** 1 Department of Orthopedics, 961th Hospital of PLA, Qiqihar, China; 2 Department of Orthopedics, 960th Hospital of PLA, Jinan, China

**Keywords:** cauda equina syndrome, complication prevention, lumbar spine infection, pressure injury, pressure sore, Vacuum Sealing Drainage

## Abstract

This report presents a complex case of a 65-year-old male patient with a lumbar spine infection (T12-L1) caused by *Staphylococcus aureus*, leading to cauda equina syndrome, and subsequently developing a massive infectious pressure sore in the sacrococcygeal region. Back pain due to spinal infections, analgesic use, neurological deficits, and systemic infections masked the early signs of the pressure sore. Prolonged immobilization and neurogenic bladder and bowel incontinence led to a 5 cm × 5 cm deep pressure sore in the sacrococcygeal area. Involving a single-stage surgery for debriding the spinal lesion and performing internal fixation, along with debriding the sacrococcygeal ulcer using Vacuum Sealing Drainage (VSD) in both infected areas, this treatment not only aids in infection control but also reduces the overall treatment duration. Through multiple VSD changes, antimicrobial therapy, and nutritional support, the spinal infection was cured, and the pressure sore wound healed. This case underscores the importance of early prevention and identification of sacrococcygeal ulcers in patients with lumbar spine infections and neurological deficits to prevent the occurrence and progression of this severe complication.

## Introduction

Pyogenic infection of the lumbar spine is a severe spinal condition that often leads to intense pain, neurological impairment, and systemic infection symptoms. When combined with cauda equina syndrome, patients are frequently confined to prolonged bed rest due to pain and neurological dysfunction, severely limiting their mobility (1). In this context, the sacrococcygeal region, as the primary pressure point in the supine position, is highly susceptible to pressure injuries (pressure sores) (2–4). Once developed, neurogenic bladder and bowel incontinence significantly increase the risk of wound contamination and infection, making treatment more challenging (5). However, current literature on sacrococcygeal pressure sores in the presence of lumbar spinal infection combined with cauda equina syndrome is scarce, and there is very limited information available regarding its treatment. This report discusses a successfully treated complex case, aims to highlight the clinical importance of early recognition and integrated management of sacrococcygeal pressure sores in patients with lumbar spinal infection complicated by cauda equina syndrome.

## Case presented

### Medical history and examination

**Figure 1:**
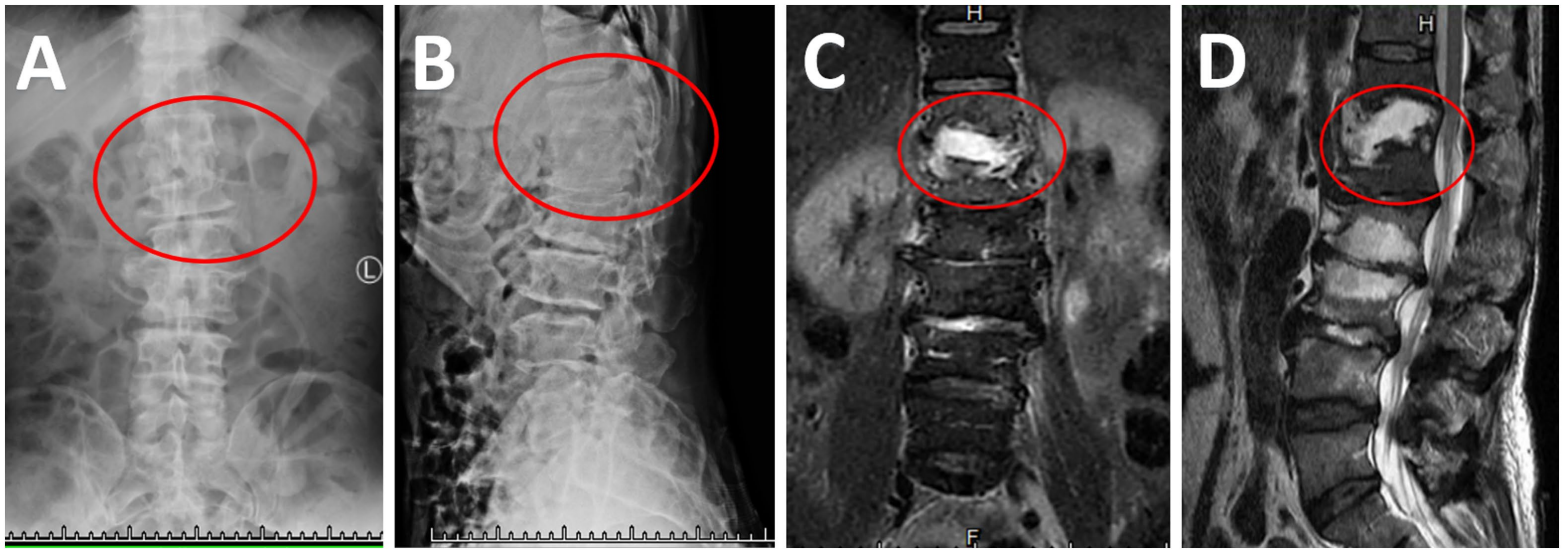
Imaging evidence of T12/L1 infection. **(A,B)** Indicate osteolysis of the T12 and L1 vertebral bodies on X-ray. **(C,D)** Show abnormal signal intensity in the T12 and L1 vertebral bodies and intervertebral disc on MRI, indicating pyogenic infection.

A 65-year-old male patient was admitted on March 12, 2025, due to “fever accompanied by back pain, and lower limb weakness for 2 months.” Initially misdiagnosed with “bacterial pneumonia” at another hospital, blood culture revealed *Staphylococcus aureus*. Upon admission, the physical examination revealed tenderness and percussion pain in the thoracolumbar spine, impaired muscle strength in both lower limbs (grade 4 on the right, grade 3 on the left), diminished sensation in the saddle area, urinary retention due to bladder dysfunction necessitating a urinary catheter, and relaxation of the anal sphincter with muscle strength graded at level 2 on the Oxford Scale. Upon admission, the patient was diagnosed with lumbar instability and cauda equina syndrome. Skin pressure marks were noted in the sacral area, which later developed into a progressively enlarging wound. Ten days later, a specialized examination revealed a stage IV pressure sore with an infected sinus tract measuring approximately 5 cm x 5 cm in the sacrococcygeal region extending to the sacrum. Radiographic findings included osteolysis of the T12 and L1 vertebral bodies on X-ray (Figures 1A,B) and abnormal signal intensity in the T12 and L1 vertebral bodies and intervertebral disc on MRI indicative of pyogenic infection (Figures 1C,D). Laboratory results showed an erythrocyte sedimentation rate of 93 mm/h and highly sensitive C-reactive protein level of 87.58 mg/L.

### Diagnosis

Spinal infection (T12, L1 vertebral interspace, *Staphylococcus aureus*); sepsis; cauda equina syndrome; pressure injury (stage IV sacral pressure sore).

### Treatment process

The core challenge of treatment lies in the conflicting needs of controlling the spinal infection through relative immobilization and fixation, while promoting pressure sore healing requires thorough decompression and avoidance of sustained pressure. The treatment plan included:

Preoperative preparation: Administer vancomycin based on sensitivity testing for infection control, and provide enteral and parenteral nutrition support to enhance overall health. Following discharge, the patient should continue taking oral linezolid tablets for 4 weeks, resulting in a total antibiotic usage duration of 11 weeks.

**Figure 2:**
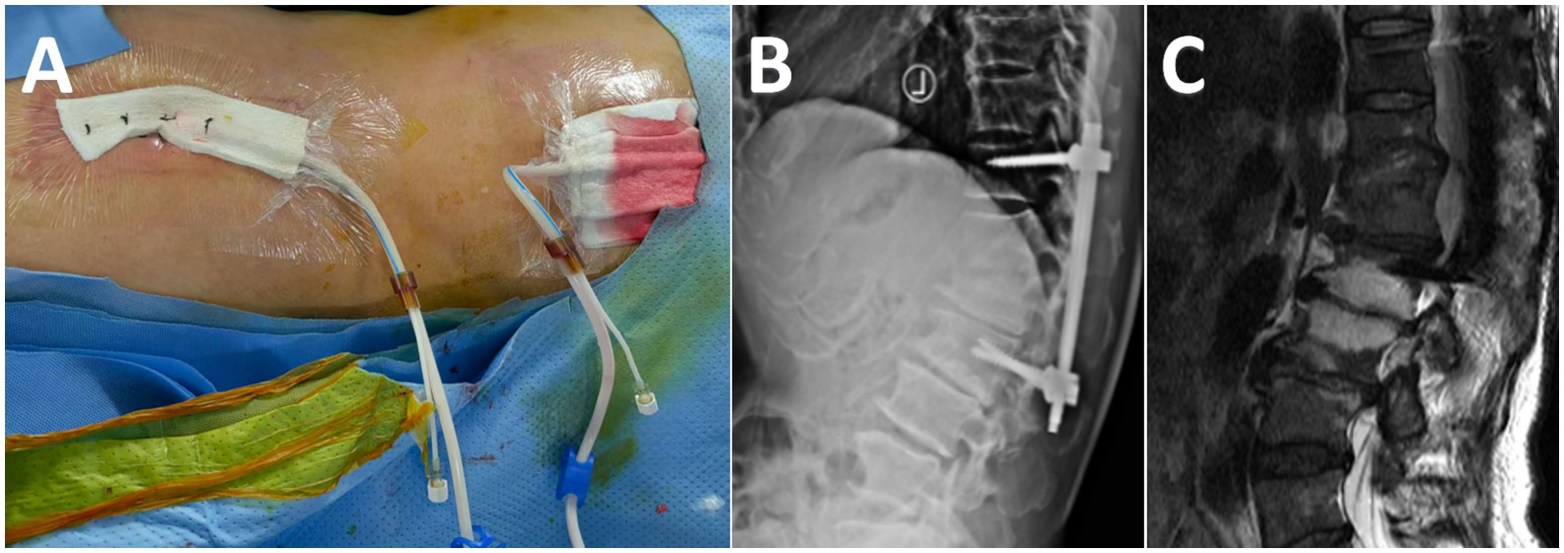
**(A)** Shows the appearance of postoperative dual-zone VSD drainage. **(B)** Indicates good fixation after surgery. **(C)** Shows the disappearance of the intervertebral abscess and inflammatory signal in the vertebral body on MRI 1 month after surgery.

Surgical intervention: Under general anesthesia, procedures included “T11-L2 pedicle screw fixation + excision of lesion at T12-L1 vertebral interspace with VSD drainage + excision of sacral pressure sore lesion with VSD drainage.” Two independent VSD devices were placed simultaneously in the extensive wounds post debridement of the spinal infection area and sacral region, creating a “dual-zone VSD drainage” for continuous and closed drainage of deep-seated infection foci (Figure 2A). The negative pressure for suction ranged from -125 mmHg to -200 mmHg, and multiple purse-string sutures and skin traction techniques were applied to address the significant skin defects in the pressure sore region.

Postoperative management: Continued administration of vancomycin for infection control. The sacral VSD provided effective drainage while maintaining a moist wound environment and promoting granulation tissue growth. A total of 7 VSD device changes were performed sequentially (42 days), adjusting VSD materials and positions based on wound conditions. Nutritional support, limb function rehabilitation, and specialized pressure sore care (including regular repositioning, use of advanced pressure-relieving cushions) were emphasized.

### Outcome

**Figure 3:**
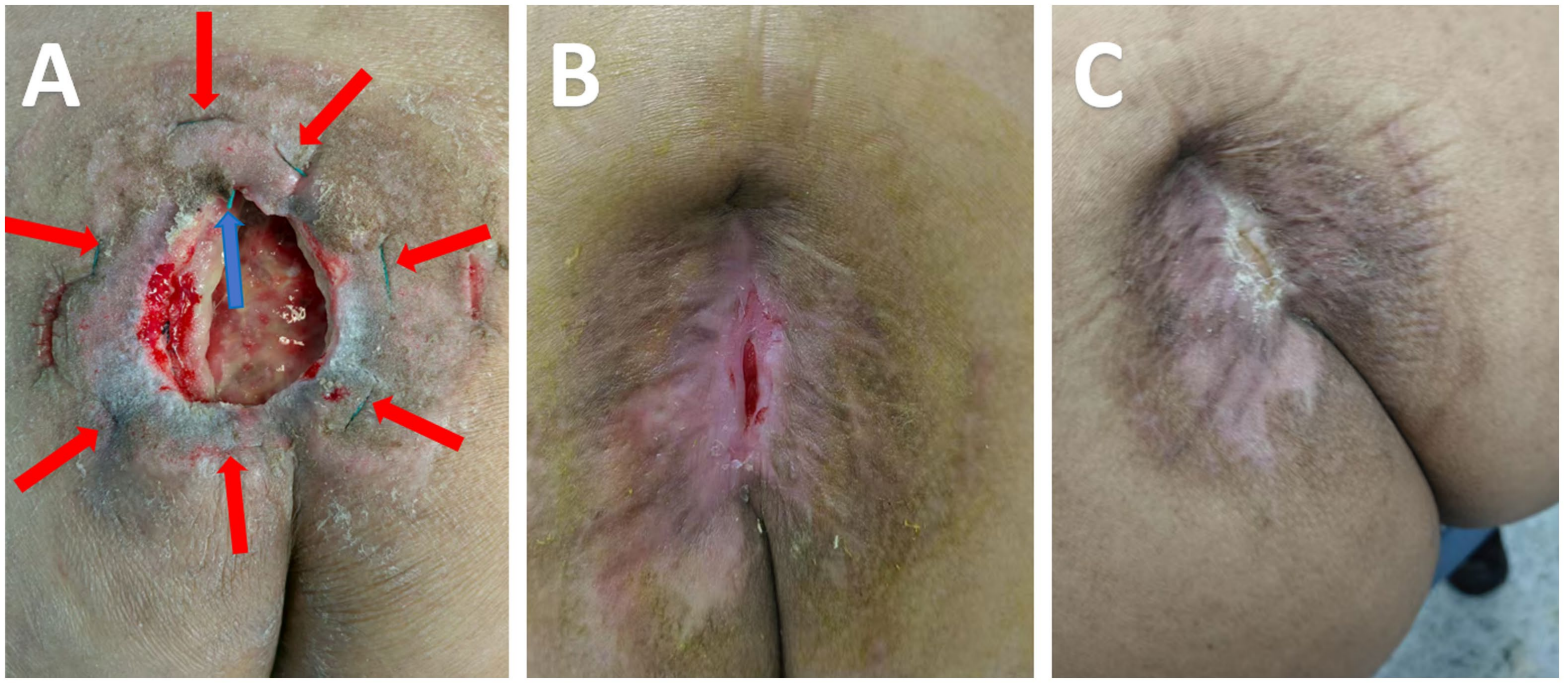
The gradual reduction of the pressure sore wound until it heals. The red arrow in **(A)** points to the green suture line outside the skin, while the blue arrow indicates the suture knot inside the skin, showing the application of the purse-string suture technique for skin traction. **(B,C)** indicates that the wound gradually shrinks until it completely heals.

After surgery, there has been partial neurological recovery. Sensation in the saddle area has improved compared to before. Bladder function has also improved, but difficulty in urination persists, requiring catheterization once every 20 days. The anal sphincter muscle strength, graded as level 4 on the Oxford Scale, remains. Bowel movements are mostly normal. Blood sedimentation and C-reactive protein have returned to normal levels, with a C-reactive protein level of 0.98ug/ml, WBC count of 3.92*10^9/L, and blood sedimentation rate of 28 mm/H. Follow-up imaging showed good position of internal fixation, resolution of spinal infection (Figures 2B,C). Excellent granulation tissue growth in the sacral wound post sequential VSD therapy, combined with tension reduction, led to gradual wound contraction and eventual healing (Figure 3). With 49 days of treatment completed, the patient was discharged, demonstrating excellent healing of the lumbar incision. During the 9-month follow-up after surgery, there was no instability in the lumbar spine. Sensation and muscle strength in both lower limbs were normal. There was a slight sensory impairment in the saddle area and the anal sphincter muscle strength was at level 4. Bowel movements were normal, but there was slight difficulty in urination, requiring catheterization once every 20 days.

## Discussion

This case vividly illustrates the vicious cycle between lumbar spine infection, especially when accompanied by nerve damage, and sacral pressure sores: pain and neurological impairments force the patient to prolonged bed rest, reducing mobility (6); systemic infection symptoms and analgesics may mask early pain and discomfort of pressure sores; cauda equina syndrome leads to urinary and fecal incontinence, severely contaminating the sacral skin, making it macerated, ulcerated, and greatly increasing the risk of bacterial invasion. Once pressure sores form and penetrate deeply to the bone, they become another stubborn source of infection, interacting with spinal infections, complicating antimicrobial therapy. The paradox of “immobilization versus activity” in treatment demands an integrated approach by clinicians. The key to success in this case was the adoption of a comprehensive surgical strategy of “primary spinal stability and lesion debridement + dual-zone vacuum-sealed drainage (VSD).” The application of dual-zone VSD achieved: simultaneous drainage, providing continuous effective drainage for deep infections in both the spine and sacral region, rapidly controlling local infections (7); and cross-contamination isolation, reducing the risk of bacterial cross-contamination between the two infection sites. There are multiple studies reporting the efficacy of VSD in treating primary spinal infections, confirming its clinical effectiveness (8, 9).

To the best of our knowledge, there are no reported cases of treating pressure sores solely through skin traction. This may be due to the fact that pressure sores commonly occur at bony prominences (such as the sacrum and coccyx), where the local skin has poor mobility and high tension (10), making it difficult to effectively apply and secure traction devices. Moreover, the surrounding tissues of pressure sores often exhibit ischemia, fibrosis, and inflammation, leading to poor elasticity. Traction in such cases can further damage blood flow or cause tissue tears, thus delaying healing (11). Since pressure sores typically involve full-thickness tissue loss with deep infections or necrosis, simple skin traction cannot address issues like deep dead spaces or vascular reconstruction, resulting in limited overall healing effects (12). In our case study, we used a combination of skin traction and VSD to stimulate granulation tissue growth (13). Additionally, we employed several wound pouch sutures to decrease the wound size (14). This strategy effectively promoted wound healing, eliminating the necessity for skin flaps or grafts and thereby shortening the treatment period (15).

The key alert in this case is that for patients with lumbar spine infection and lower limb neurological symptoms, pressure sore prevention must be a core component of the initial treatment phase, rather than a remedial measure. Early prevention strategies should include: 1. Risk assessment and alert, using tools like the Braden Scale upon admission for systematic evaluation to categorize these patients as extremely high-risk for pressure sores (16); 2. Systematic pressure relief measures, developing a strict repositioning schedule (e.g., every 2 h) for patients tolerating it, even during periods of significant pain, axial rotation should be done under healthcare provider guidance using specialized tools like air mattresses, pressure-relief cushions, etc. (17); 3. Early skin monitoring and fecal incontinence management, conducting comprehensive daily skin checks, especially in areas with reduced sensation like the sacrum, for patients with fecal incontinence, strict preventive measures for incontinence-related dermatitis are necessary, including using highly absorbent pads, prompt cleansing, and application of skin protectants (18).

## Conclusion

Patients with lumbar spine infection complicated by cauda equina nerve damage are at high risk of developing severe sacral pressure sores. It is extremely important to prevent their occurrence early. The utilization of debridement and internal fixation in the acute lesion area, along with dual-zone VSD drainage, can effectively manage the infection, promote wound repair, and reduce the treatment duration.

## Data Availability

The original contributions presented in the study are included in the article/supplementary material, further inquiries can be directed to the corresponding authors.
